# Cross-sectional psychometric validation, convergent validity, and measurement invariance of the DASS-21 in Mexican university students

**DOI:** 10.3389/fpsyg.2025.1707786

**Published:** 2025-12-08

**Authors:** Luis Hernando Silva Castillo, Enrique Hernández-Rosas, Juan Carlos Silas-Casillas, Sarah Nayibe Cazares, Luis Carlos Fonseca León

**Affiliations:** 1 Instituto Tecnológico y de Estudios Superiores de Occidente (ITESO), Tlaquepaque, Mexico; 2 Posgrado en Ciencias del Comportamiento, Universidad de Guadalajara, Guadalajara, Mexico

**Keywords:** depression anxiety stress scales, psychometric validation, reliability, Mexican university students, coping styles, bifactor model

## Abstract

**Objective:**

To evaluate the factor structure, reliability, and gender invariance of the DASS-21, and to examine convergent/criterion-related evidence with coping and functional impairment.

**Design:**

Cross-sectional, single-site psychometric validation in first-year undergraduates at a private Mexican university; multigroup CFA with ordinal/WLSMV; proctored digital administration; convergent/criterion analyses and known-groups tests.

**Methods:**

First-year students (*N*=1,251) completed the DASS-21 and an adapted Proactive Coping Inventory. Models estimated from polychoric correlations (WLSMV) compared a correlated three-factor solution, a second-order model, and a bifactor model; ESEM served as a robustness check. Gender invariance (women/men) followed a configural–metric–scalar (threshold) sequence. Associations with coping, prior diagnosis, and recent functional impairment were tested with multiplicity control.

**Results:**

The three-factor model showed acceptable fit (CFI=0.99; RMSEA=0.07). Hierarchical/bifactor solutions improved representation; bifactor indices (ECV=0.81; ωh=0.88) supported a predominant general factor. Reliability was good–excellent (ωsubscales≈0.85–0.88; ωtotal≈0.94). Configural, metric, and scalar (threshold) invariance held (ΔCFI≤0.01; ΔRMSEA≤0.015). Latent means indicated higher Stress and Anxiety in women (small effects), with no difference in Depression. DASS-21 scores correlated negatively with adaptive coping and positively with avoidant coping; higher scores were observed among students reporting prior diagnosis and recent functional impairment.

**Conclusion:**

Evidence supports the validity, reliability, and fairness of the DASS-21 for screening general distress and profiling subdomains in Mexican universities; priorities include norms and ROC-based cutoffs in verified clinical samples. Limitations: Single-site, non-probabilistic sample; cross-sectional design; clinical status by self-report; forced-completion digital setting may introduce minor response pressure.

## 1 Introduction

University students’ mental health has become a critical concern in recent years, particularly in Mexico. Even before the COVID-19 pandemic, considerable prevalence rates of anxiety (19%–31%) and depression (12%–25%) had been reported among young adults (INEGI, 2021; Vázquez-Salas et al., 2023). During and after the pandemic, these rates increased alarmingly, with more than two-thirds of students reporting anxiety symptoms and over half reporting depressive symptoms (Hernández-Fuentes et al., 2024). Factors such as social isolation, economic uncertainty, academic overload, and unequal access to mental-health services have exacerbated this crisis, with particularly high impact on women and students from middle- and lower-socioeconomic backgrounds (Stallman, 2010; Beiter et al., 2015). Emotional symptoms in emerging adults arise from ongoing goal–environment appraisals and the coping responses mobilized to manage perceived demands (Lazarus and Folkman, 1984).

In this context, brief and culturally relevant instruments are essential for the timely identification of emotional symptoms in university populations. Although numerous tools exist to measure depression, anxiety, or stress in isolation, few allow for a simultaneous, differentiated, and efficient assessment. The Depression, Anxiety, and Stress Scales–21 items (DASS-21; Lovibond and Lovibond, 1995) stands out as a robust alternative: grounded in Clark and Watson’s (1991) tripartite model, it assesses three interrelated yet distinct emotional syndromes and has shown solid psychometric properties across diverse countries and populations (Antony et al., 1998; Henry and Crawford, 2005). Within this framework, examining DASS-21 scores alongside coping resources is theoretically coherent, as stress (sustained tension), anxiety (anticipatory arousal), and depression (diminished positive affect) represent downstream outcomes of appraisal and coping processes within the *transactional model*.

International and Latin American studies consistently support the reliability and validity of the DASS-21, including validations in Chile (Antúnez and Vinet, 2012; Román et al., 2014), Colombia (Ruiz et al., 2017), and Spain (Fonseca-Pedrero et al., 2010). Evidence on the DASS-21 in Mexico remains heterogeneous. While existing studies have contributed to mapping the scale’s dimensionality, key gaps persist regarding fair subgroup comparisons and external interpretability. Early work proposed shortened versions (e.g., *DASS-14*) through item deletion without confirmatory modeling or invariance testing, which limited replicability and construct coverage (Gurrola-Peña et al., 2006). During the COVID-19 lockdown, a national-sample analysis compared structural alternatives and favored a bifactor representation dominated by a general distress factor; however, multi-group invariance and convergent validity with external measures were not reported, leaving issues of comparability and interpretability beyond model fit unresolved (Salinas-Rodríguez et al., 2023). In smaller university samples, the three-factor model achieved very high fit indices—likely inflated by sample size and model specification—yet without testing invariance or convergent/discriminant validity, which limits generalization (Salinas-Muñoz et al., 2024). In community-dwelling adults, ESEM and bifactor-ESEM approaches supported brief forms (e.g., *DASS-12*) and confirmed a strong general factor; nevertheless, these studies omitted invariance testing and external validation (e.g., coping, functional impairment), emphasizing structural adequacy over comparability and practical interpretability (Domínguez-Lara et al., 2024). Finally, occupational studies applied the 21-item version in worker samples but did not address invariance or convergent validity, leaving the transferability to university contexts and fairness of comparisons uncertain (Cruz-Flores et al., 2024).

From a theoretical perspective, the *transactional model of stress and coping* (Lazarus and Folkman, 1984) conceptualizes psychological distress as the result of the dynamic interaction between environmental demands and individual coping resources. Within this framework, emotions such as stress, anxiety, and depression assessed by the DASS-21 represent outcomes of appraisal and coping processes. Coping functions as a regulatory mechanism that determines how individuals interpret, manage, and recover from stressful experiences. Consequently, assessing coping resources alongside indicators of distress provides a more comprehensive understanding of emotional adjustment. The Proactive Coping Inventory (PCI; Greenglass et al., 1999) extends this model by emphasizing anticipatory and goal-oriented coping capacities that buffer stress and promote adaptation, offering a theoretically coherent basis for examining convergent validity with the DASS-21.

The decision to evaluate measurement invariance across gender rests on both theoretical and empirical grounds. Group differences in DASS-21 scores may reflect measurement bias rather than true latent variation; therefore, establishing configural, metric, and scalar (threshold) invariance is required for meaningful comparisons and aligns with international standards for validity and fairness (American Educational Research Association, American Psychological Association, and National Council on Measurement in Education, 2014; European Federation of Psychologists’ Associations, 2025). Empirically, Mexican and Latin American studies frequently report higher scores among women without verifying whether these reflect latent disparities or biased item functioning (e.g., Salinas-Rodríguez et al., 2023; Cruz-Flores et al., 2024). Only one study has tested gender-based invariance—and on shortened adult forms (*DASS-12*, *DASS-8*), not the full DASS-21 (Domínguez-Lara et al., 2024). Accordingly, the present study provides new evidence by evaluating invariance of the complete DASS-21 across gender in a large university sample, ensuring interpretable cross-group comparisons.

This study aimed to address these gaps by validating the DASS-21 in a large, single-site sample from a private Mexican university. The factorial structure, internal consistency, and gender invariance were examined, together with convergent validity using the Proactive Coping Inventory (Greenglass et al., 1999). In addition, by reporting bifactor structure indices (e.g., *ECV*, *ωₕ*, *PUC*) alongside multigroup tests, this study complements existing Mexican evidence based on ESEM and bifactor-ESEM brief forms by enhancing comparability and external interpretability. This approach strengthens the usefulness of the DASS-21 as a reliable and culturally appropriate instrument for screening symptoms of depression, anxiety, and stress in university contexts. Although several Mexican studies have examined the DASS-21, most relied on small, homogeneous samples and did not test measurement invariance or convergent validity; the current research addresses these limitations. Based on previous empirical evidence and theoretical models, the following hypotheses were formulated:

*H*1a (Three-factor structure): The DASS-21 will exhibit a correlated three-factor structure (stress, anxiety, depression) with acceptable global fit.

*H*1b (General factor predominance): Bifactor or second-order evaluations will indicate a prominent general distress factor (e.g., high *ECV* and *ωₕ*), while retaining interpretable specific factors.

*H*2 (Reliability): Internal consistency will be adequate to excellent (*α* and *ω* ≥ 0.80 for subscales; ≥ 0.90 for the total score), with narrow confidence intervals indicating stable estimates.

*H*3 (Measurement invariance by gender): The three-factor model will demonstrate configural, metric, and scalar (threshold) invariance across women and men under an ordinal (WLSMV) framework, with negligible decrements in fit at each step (e.g., Δ*CFI* ≤ 0.01; Δ*RMSEA* ≤ 0.015).

*H*3a (Latent mean differences; conditional on H3): Given scalar (threshold) invariance, women will present higher latent means on Stress and Anxiety than men (small effects), with no meaningful difference on Depression.

*H*4 (Convergent validity with coping): DASS-21 scores (total and subscales) will correlate negatively with adaptive coping styles (Social Support, Reflective/Preventive Coping, Proactive Coping, Strategic Planning) and positively with Avoidant/Evasive coping. The strongest association is expected with Avoidant/Evasive coping. Statistical significance will be evaluated using Holm’s procedure to control familywise error.

*H*5 (Known-groups validity): Students reporting a prior clinical diagnosis of anxiety and/or depression will obtain higher DASS-21 total and subscale scores than those reporting no diagnosis.

*H*6 (Criterion-related validity: functional impairment): Higher DASS-21 scores will be associated with greater recent functional impairment (e.g., doing less than intended, stopping activities); the total score is expected to show the strongest associations.

## 2 Materials and methods

### 2.1 Participants

The sample consisted of *N* = 1,251 first-year undergraduate students enrolled in diverse academic programs at a single private university in Mexico. Because the data were obtained from a single-site private institution, claims of population heterogeneity are not warranted, and generalizability should be interpreted cautiously. Inclusion criteria were (a) being a first-year undergraduate student officially enrolled during the 2024–2025 academic year and (b) providing informed consent. Exclusion criteria included incomplete responses or declining consent to participate. As part of the sociodemographic questionnaire (see Measures), participants answered a single self-report item on prior mental-health diagnoses. This item was used solely to contextualize the sample and to derive a binary grouping variable (clinical: Depression or Anxiety vs. non-clinical: None) for known-groups validity analyses (see Section 3.4).

Regarding gender identity, 50.3% identified as men, 48.5% as women, 0.4% as non-binary, 0.1% as other identities, and 0.7% preferred not to answer. The mean age was 18.65 years (*SD* = 1.40). However, 17.5% of responses for this variable were missing due to a data-entry error in the digital form, where some participants recorded only their birth day and month instead of the complete date of birth. Concerning marital status, 81.4% reported being single and 16.8% in a relationship.

In terms of physical activity, 34.6% reported exercising more than three times per week, 25.2% two or three times, 16.8% daily, 14.3% once a week, and 9.1% never. Regarding self-reported indicators of impairment during the previous week, 8.7% of participants reported doing less than desired due to physical reasons, and 4.2% indicated that they had to stop daily activities for the same cause. Emotionally, 25.4% reported doing less than desired and 12.6% had to suspend activities due to emotional distress. A detailed summary of sociodemographic, contextual, and impairment indicators is presented in Table 1.

**Table 1:** Sociodemographic, contextual characteristics, and indicators of impairment in the sample.

Variable	Category	*n*	%	Missing, *n* (%)
Gender identity	Male	629	50.3	—
	Female	607	48.5	—
	Non-binary	5	0.4	—
	Other	1	0.1	—
	Prefer not to say	9	0.7	—
Marital status	Single	1,018	81.4	—
	In a relationship	210	16.8	—
	Prefer not to answer	20	1.6	—
	Other	3	0.2	—
Frequency of physical activity	More than three times/week	433	34.6	—
	Two or three times/week	315	25.2	—
	Daily	210	16.8	—
	Once a week	179	14.3	—
	Never	114	9.1	—
Indicators of physical impairment (last week)	Did less than desired	109	8.7	2 (0.2)
	Had to stop daily activities	53	4.2	2 (0.2)
Indicators of emotional impairment (last week)	Did less than desired	318	25.4	2 (0.2)
	Had to stop daily activities	158	12.6	4 (0.3)

*n* = frequency. Percentages are based on valid responses. Missing data are reported in the corresponding column. Impairment indicators refer to the previous week.

The sample size (*N* = 1,251) provides substantial statistical power for the intended confirmatory factor analyses and multigroup invariance testing. According to methodological reviews (Kyriazos, 2018; Kline, 2016; Brown, 2015), samples above 200 are considered adequate for CFA, whereas 500–1,000 are deemed very good to excellent (Comrey and Lee, 1992). Moreover, simulation studies for ordinal data analyzed with robust estimators (e.g., WLSMV) recommend between 200 and 500 participants per group (Bandalos, 2014). Therefore, the present sample largely exceeds established recommendations, ensuring stable parameter estimation and reliable fit indices.

### 2.2 Procedure

This cross-sectional, single-site psychometric validation collected data digitally between August 12 and 18, 2024, in the university’s computer labs, each equipped with 50 workstations. To ensure standardized conditions, test administrators received 2 h of training and followed a written protocol available throughout the sessions. Environmental conditions (lighting, temperature, and noise) were controlled, and participants were seated to maintain physical separation. The instruments were administered through the SurveyMonkey^®^ platform, which presented items and sociodemographic questions in randomized order.

Prior to participation, students received information about the study objectives and provided written informed consent. They were assured of confidentiality, the exclusive use of data in aggregated form, and the right to withdraw at any point without academic consequences. No incentives were offered. Data were downloaded with random identifiers, ensuring pseudonymization and preventing any linkage to individuals.

As part of the digital assessment, participants also completed a brief sociodemographic questionnaire that included an item regarding prior mental health diagnoses. The question asked: “Have you ever been diagnosed with any of the following conditions?” with response options: None, Depression, Anxiety, Attention-Deficit Disorder (with or without hyperactivity), Autism spectrum-related condition, and other (please specify). This self-report item was included to contextualize the sample and evaluate discriminant validity. It reflects participants’ own reports of previous professional evaluations; no structured clinical interview or diagnostic verification was conducted as part of this study.

The study was approved by the institutional ethics committee (approval code: 072-CEI08-2024). The version of the Depression Anxiety Stress Scale–21 items (DASS-21) used in this study was based on the Mexican adaptation by Salinas-Muñoz et al. (2024), which had been previously revised for semantic and conceptual equivalence to the original version. A panel of four experts (two clinical psychologists with experience in university populations and two PhD-level psychologists specialized in measurement and test construction) reviewed the items in a group session, assessing clarity, relevance, and cultural appropriateness. Minimal wording adjustments were made following their recommendations. The preliminary version was piloted with 41 first-year students to evaluate comprehension and administration time, after which final adjustments were implemented, yielding the version analyzed in this study.

### 2.3 Instruments

#### 2.3.1 Depression anxiety stress scale–21 items (DASS-21)

The DASS-21 (Lovibond and Lovibond, 1995) is a self-report instrument designed to assess symptoms of depression, anxiety, and stress during the past week. For the present study, the Mexican adaptation by Salinas-Muñoz et al. (2024) was used, which builds on previous validations conducted in Spain (Bados et al., 2005) and Colombia (Salinas-Rodríguez et al., 2023).

The scale consists of 21 items divided into three subscales of seven items each: Depression (e.g., anhedonia, hopelessness, self-devaluation), Anxiety (e.g., autonomic arousal, situational anxiety, subjective anxious affect), and Stress (e.g., difficulty relaxing, irritability, nervous tension). Responses are recorded on a 4-point Likert-type scale ranging from 0 (*Did not apply to me at all*) to 3 (*Applied to me very much or most of the time*). Subscale scores are obtained by summing the item responses (range = 0–21), and the total score represents the sum of all 21 items (range = 0–63). Although scores can be doubled to maintain comparability with the original 42-item version, raw (non-doubled) scores were analyzed in this study. The full Spanish adaptation, including original wording, item modifications, and administration/scoring notes, is provided in Supplementary Table S1 (Section A).

Previous research has reported a stable three-factor structure and high reliability (*α* and *ω* ≥ 0.80). Among Mexican university students, Salinas-Muñoz et al. (2024) reported excellent model fit (*CFI* = 1.00, *TLI* = 1.00, *RMSEA* = 0.000) and high internal consistency (*α* = 0.83–0.90; *ω* = 0.84–0.90).

#### 2.3.2 Proactive coping inventory – adapted version (PCI-VA)

The Proactive Coping Inventory (PCI; Greenglass et al., 1999) assesses strategies for managing stressors. The adapted version used in this study was developed by the research team based on the original instrument and the adaptation guidelines proposed by Greenglass et al. (1999), and it was designed for application in Mexican university students. This version includes five dimensions: Seeking Social Support, Reflective/Preventive Coping, Proactive Coping, Avoidant Coping, and Strategic Planning. Items are rated on a 4-point Likert-type scale. Subscale and total scores are obtained by summing the responses.

In the present sample, internal consistency indices were satisfactory. Cronbach’s alpha (*α*) and McDonald’s omega (*ω*) coefficients for each subscale were as follows: Strategic Planning (*α* = 0.70, *ω* = 0.75), Avoidant Coping (*α* = 0.77, *ω* = 0.79), Instrumental/Emotional Support (*α* = 0.90, *ω* = 0.90), Proactive Coping (*α* = 0.87, *ω* = 0.88), and Reflective/Preventive Coping (*α* = 0.89, *ω* = 0.89). Ninety-five percent confidence intervals (bootstrap, 1,000 iterations) indicated robust reliability across subscales (*α* = [0.62–0.95]; *ω* = [0.69–0.97]). These results confirm the internal consistency of the PCI within this university sample. Reliability indices for the Proactive Coping Inventory used in the convergent-validity analyses are presented in Supplementary Table S11.

Exploratory and confirmatory factor analyses supported its structural validity, indicating satisfactory model fit (*χ*^2^ = 2051.64, *df* = 892, *p* < 0.001; *RMSEA* = 0.060; *CFI* = 0.978; *TLI* = 0.977; *GFI* = 0.969). In this study, the PCI-VA served as an external criterion for assessing the convergent validity of the DASS-21.

#### 2.3.3 Sociodemographic questionnaire

An *ad hoc* sociodemographic and contextual questionnaire was developed to characterize the sample. It collected demographic variables (age, gender identity, marital status, academic program, place of origin, family living arrangements, sources of income, and scholarship or institutional support), health-related habits (physical activity; use of tobacco, alcohol, and other substances), and indicators of physical and emotional impairment during the past week.

This questionnaire also included a single self-report item on prior mental-health diagnoses: *“Have you ever been diagnosed with any of the following conditions?”* Response options were *None, Depression, Anxiety, Attention-Deficit Disorder (with or without Hyperactivity), Autism spectrum–related condition,* and *Other (please specify).* This item was incorporated to contextualize the sample and to support discriminant-validity analyses.

Because the instrument was designed for descriptive purposes rather than for the measurement of a latent construct, no reliability coefficients were estimated. Responses were analyzed descriptively, and no structured clinical interview or diagnostic verification was conducted.

### 2.4 Data analysis

All analyses were conducted in R (4.5.1; R Core Team, 2024) using RStudio (Posit Team, 2025). Latent-variable models were fit with lavaan (0.6–20; Rosseel, 2012) and semTools (0.5–7; Jorgensen et al., 2025); psych (2.5.6; Revelle, 2025) supported psychometric computations; and the tidyverse facilitated data wrangling and visualization (Wickham et al., 2019). Because DASS-21 items are ordinal, models were estimated from polychoric correlation matrices using the DWLS/WLSMV estimator with robust standard errors and scaled test statistics. No missing data were present because the survey platform required complete responses before submission.

Item- and scale-level descriptive statistics were computed (*M*, *SD*, skewness, kurtosis, range); response distributions were inspected, and corrected item–total correlations were examined (criterion ≥ 0.30). Due to space constraints, complete descriptive tables are reported in the Supplementary material.

For structural validity, a theory-driven family of models was estimated beginning with a correlated three-factor CFA (Depression, Anxiety, Stress). Global fit was evaluated using *χ*^2^, *CFI*, *TLI*, *RMSEA*, and *SRMR* (acceptable: *CFI/TLI* ≥ 0.90; close fit ≥ 0.95; *RMSEA* < 0.08 [good < 0.05]; *SRMR* < 0.08).

To probe the balance between general and specific variance, a second-order hierarchical model (three first-order factors loading on a general distress factor) and a bifactor model (one general factor plus three orthogonal specific factors) were estimated. For the bifactor solution, the general/specific variance partition was quantified using *ECV*, *ωₕ*, and *PUC* (computed with semTools/psych). In line with recommendations on cross-loadings, an ESEM model with Geomin rotation was additionally estimated as a robustness check; full ESEM diagnostics (fit indices and loadings) are provided in the Supplementary material.

Reliability was evaluated using McDonald’s *ω* and Cronbach’s *α* (good 0.70–0.90; excellent > 0.90), with 95% bootstrap confidence intervals (1,000 resamples) where applicable (e.g., PCI subscales).

Measurement invariance was assessed across gender (women/men) using a hierarchical sequence under the ordinal (WLSMV) framework: configural, metric, and scalar (threshold) invariance. Decisions were based on Δ*CFI* ≤ 0.01 and Δ*RMSEA* ≤ 0.015 (Δ*SRMR* reported for reference). The non-binary subgroup (*n* = 5) was analyzed descriptively due to insufficient power for stable multigroup estimation.

Conditioned on scalar (threshold) invariance, latent means were compared by fixing the reference-group mean to zero and estimating *ΔM* (with 95% CIs) for the comparison group on each latent factor (Stress, Anxiety, Depression). Effect magnitudes were interpreted in the small-to-moderate range given the standardized latent metric (std.lv = 1).

Associations between DASS-21 dimensions (total and subscales) and PCI-VA coping factors were analyzed using Spearman’s *ρ* (two-tailed, *α* = 0.05). To control multiplicity, *p*-values were Holm-adjusted by families (Total × PCI; Stress × PCI; Anxiety × PCI; Depression × PCI) using Holm’s step-down procedure for familywise error control.

Group differences were examined between participants with versus without a self-reported prior diagnosis of anxiety/depression using Welch’s *t* tests or Mann–Whitney *U*-tests, as appropriate given distributional assumptions. Effect sizes were reported as Hedges’ *g* (Welch’s *t*) and rank-biserial *r* (Mann–Whitney), with 95% confidence intervals.

Associations between DASS-21 scores and recent functional impairment (e.g., doing less than intended; stopping activities) were evaluated using linear/ordinal models or logistic regression, depending on outcome scaling. When multiple impairment criteria were modeled within a family of tests, Holm’s procedure was applied for multiplicity control.

Potential confounders (e.g., socioeconomic status, academic program) were recorded and inspected descriptively but not modeled as covariates, as the study focused on psychometric validation rather than prediction.

### 2.5 Ethics statement

The study was reviewed and approved by the research ethics committee (Approval No. 072-CEI08-2024, August 2024), in accordance with the principles of the *Declaration of Helsinki* (World Medical Association, 2013) and the *Regulations of the General Health Law on Health Research* (Mexico) (Secretaría de Salud, 2014). All participants were individually informed about the study’s purpose and procedures and provided written informed consent, along with an additional authorization form for the use of their data for research purposes.

Participation was voluntary, and participants retained the right to withdraw at any time without consequences. Confidentiality and anonymity were ensured through pseudonymization of the dataset. That is, replacing direct identifiers with random codes and all records were securely stored with restricted access limited to the research team.

## 3 Results

### 3.1 Item-level descriptive statistics

Items ranged from *M* = 0.29 to 1.49, with standard deviations (*SDs*) between 0.69 and 1.02. The highest endorsement was observed for nervousness items—Item 8 (*M* = 1.49) and Item 11 (*M* = 1.46)—whereas the lowest mean was recorded for the hopelessness indicator, Item 21 (*M* = 0.29). Distributions were generally right-skewed (*skew* = 0.13–2.58), with leptokurtic shapes for items reflecting more severe content (e.g., Item 21: *skew* = 2.58, *kurtosis* = 6.13; Item 17: *skew* = 1.93, *kurtosis* = 2.89), consistent with infrequent endorsement in a non-clinical student sample. Somatic/panic indicators (Items 4, 7, 15, and 19) also showed pronounced asymmetry and kurtosis (e.g., Item 4: *skew* = 1.50, *kurtosis* = 1.61).

No ceiling effects were detected; a mild floor tendency appeared across low–base-rate items, supporting the use of ordinal estimators. Descriptive statistics for all 21 items (*means, SDs, skewness,* and *kurtosis*) are presented in Supplementary Table S2.

### 3.2 Confirmatory factor analysis and model comparisons

The original three-factor model of the DASS-21 was tested using confirmatory factor analysis (CFA) with the Diagonally Weighted Least Squares (DWLS) estimator on the polychoric correlation matrix, appropriate for ordinal data. The model demonstrated good overall fit to the data (*χ*^2^(186) = 1249.35, *p* < 0.001; *CFI* = 0.99; *TLI* = 0.99; *RMSEA* = 0.07; *SRMR* = 0.05). Both *CFI* and *TLI* exceeded the recommended threshold of ≥ 0.90; *RMSEA* fell within the acceptable range of 0.05–0.08, and *SRMR* met the criterion of ≤ 0.08.

**Figure 1:**
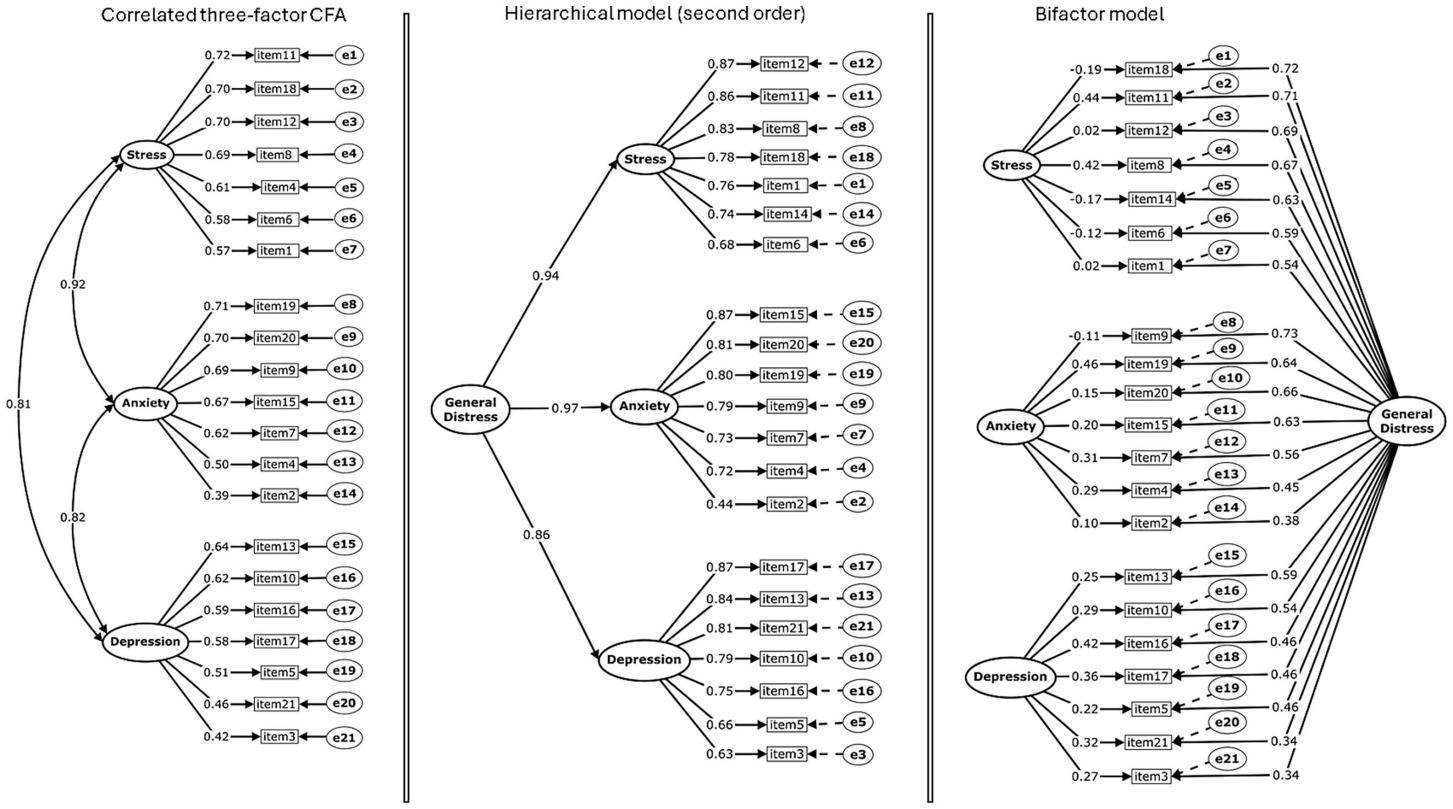
**(A)** correlated three-factor CFA. **(B)** second-order hierarchical model. **(C)** bifactor model (general factor + three specific factors). All models estimated via DWLS on a polychoric correlation matrix; standardized loadings in the main paths ranged from 0.55 to 0.86. Complete standardized loadings for the hierarchical, bifactor, and ESEM solutions are available in Supplementary Tables S5–S7 (Section C).

The chi-square statistic was significant (*p* < 0.001), as is common in large samples; therefore, interpretation prioritized robust fit indices. Standardized loadings were statistically significant (*p* < 0.001) and satisfactory (≥ 0.39). Latent-factor correlations were high—Stress–Anxiety (*r* = 0.92), Stress–Depression (*r* = 0.81), and Anxiety–Depression (*r* = 0.82)—suggesting a prominent common component alongside differentiated dimensions. The model specification and standardized loadings for the correlated three-factor solution are illustrated in Figure 1A.

Given the high inter-factor correlations, two theoretically coherent alternatives were evaluated: (a) a second-order (hierarchical) model in which the three first-order factors load on a general Distress factor, and (b) a bifactor model with one general factor and three orthogonal specific factors (Stress, Anxiety, Depression). All three models showed good fit (see Table 2).

**Table 2:** Comparative fit indices and structural reliability indicators for alternative DASS-21 models.

Model	CFI	TLI	RMSEA	SRMR	ΔCFI vs. 3F	ΔRMSEA vs. 3F	Key reliability/structure indicators
Three-factor CFA (oblique)	0.989	0.988	0.068	0.052	—	—	—
Hierarchical CFA	0.990	0.988	0.068	0.052	+0.001	0.000	Second-order loadings: Stress = 0.95; Anxiety = 0.96; Depression = 0.85 (R^2^ = 0.93, 0.96, 0.72)
Bifactor CFA	0.997	0.996	0.022	0.037	+0.008	−0.046	ECV = 0.81; ωₕ = 0.88; PUC = 0.41
ESEM (Geomin rotation)^†^	0.870	0.822	0.116	0.046	+0.003	−0.023	—

Models estimated on polychoric correlations with DWLS/WLSMV (robust). Deltas (ΔCFI, ΔRMSEA) are relative to the oblique three-factor CFA. For the hierarchical model, second-order loadings summarize the general factor contribution; for the bifactor, reliability indices include ECV (Explained Common Variance), ωₕ (omega hierarchical), and PUC. Full *χ*^2^, df, and RMSEA 90% CIs appear in Supplementary Table S4.

† ESEM was retained as a robustness check and is not directly comparable to CFA-family models.

Incremental changes relative to the correlated three-factor baseline (Δ*CFI*, Δ*RMSEA*; Table 2) favored the bifactor solution, which showed the largest improvement (*CFI* = 0.997, *RMSEA* = 0.022; Δ*CFI* = +0.008; Δ*RMSEA* = −0.046), followed by the hierarchical model (*CFI* = 0.990, *RMSEA* = 0.068; Δ*CFI* = +0.001; Δ*RMSEA* = −0.035). Bifactor indices further indicated a predominant general factor (*ECV* = 0.81; *ωₕ* = 0.88; *PUC* = 0.41). In the hierarchical model, second-order loadings were high (*λ* = 0.85–0.96; *R^2^* = 0.72–0.96), consistent with a strong common variance component.

Collectively, these results support the robustness of the DASS-21 structure, evidencing a dominant general distress continuum coexisting with three interpretable and correlated subdimensions. Fit indices for all estimated models (three-factor CFA, hierarchical, bifactor, ESEM, and the DASS-12 short form) are presented in Supplementary Table S4.

An ESEM specification with Geomin rotation was estimated as a robustness check to address potential cross-loadings. Despite this flexibility, the model showed inferior fit and unstable factor definitions compared with the CFA-family models. Likewise, results for the short form (DASS-12) are provided in the Supplementary material. Although model fit for the short form was excellent, interpretation in the present study prioritized the full DASS-21 to preserve construct coverage and ensure comparability.

### 3.3 Scale-level descriptives and reliability

Table 3 presents the *means* (*M*), *standard deviations* (*SD*), observed ranges, and reliability coefficients for the three DASS-21 subscales (Depression, Anxiety, and Stress) and the total score. The subscales demonstrated good internal consistency (*ω* = 0.85–0.88), and the total scale reached excellent values (*ω* = 0.94; *α* = 0.94). Subscale–total correlations were high (0.84–0.94), supporting the internal coherence of the instrument.

**Table 3:** Descriptive statistics (means, standard deviations, ranges), internal consistency coefficients (McDonald’s ω, Cronbach’s α), and subscale–total correlations of the DASS-21.

Subscale	*M* (*SD*)	Observed range	*ω*	*α*	Subscale–total correlation
Stress	7.9 (4.9)	0–21	0.88 [0.87–0.89]	0.88 [0.87–0.89]	0.94
Anxiety	5.4 (4.6)	0–21	0.84 [0.83–0.86]	0.85 [0.83–0.86]	0.90
Depression	4.4 (4.2)	0–21	0.85 [0.83–0.86]	0.85 [0.83–0.86]	0.84
Total	17.7 (12.4)	0–63	0.94 [0.93–0.94]	0.94 [0.93–0.94]	—

*M*, mean; SD, standard deviation; ω, McDonald’s omega (total); α, Cronbach’s alpha. Ninety-five percent confidence intervals for ω and α were estimated via nonparametric percentile bootstrap (1,000 resamples).

Descriptive statistics for each item and their corrected item–total correlations are included in the Supplementary material and are available upon request, in order to optimize manuscript space and avoid presenting a level of detail that could allow inferences about individual or group profiles within the sample. Item–total correlations and reliability estimates (Cronbach’s α, McDonald’s ω) are detailed in Supplementary Table S3 (Section B).

### 3.4 Measurement invariance

Measurement invariance across gender was tested with a hierarchical sequence of multigroup CFAs using ordinal indicators and the WLSMV estimator in *lavaan* (three correlated factors: Stress, Anxiety, Depression). Model fit indices are summarized in Table 4. The configural model (same factor structure, freely estimated parameters across groups) showed good fit (CFI = 0.99, RMSEA = 0.07, SRMR = 0.06), indicating that the three-factor structure replicated in women (*n* = 607) and men (*n* = 629). Constraining factor loadings to equality (metric invariance) produced a negligible change relative to the configural model (ΔCFI = −0.001, ΔRMSEA = +0.002, ΔSRMR = +0.003), supporting metric invariance. For scalar invariance with ordinal data, we constrained item thresholds (rather than intercepts) in addition to loadings; this model also showed no meaningful loss of fit compared to the metric model (CFI = 0.99, RMSEA = 0.06, SRMR = 0.06; ΔCFI = +0.001, ΔRMSEA = −0.005, ΔSRMR = −0.003). Collectively, these results meet common decision rules (e.g., ΔCFI ≤ 0.01; ΔRMSEA ≤ 0.015), supporting full configural, metric, and scalar (threshold) invariance across gender and thereby justifying latent-mean comparisons.

**Table 4:** Model fit indices (CFI, RMSEA, SRMR) and differences (ΔCFI, ΔRMSEA, ΔSRMR) for configural, metric, and scalar models of the DASS-21, testing measurement invariance across gender.

Step	CFI	ΔCFI	RMSEA	ΔRMSEA	SRMR	ΔSRMR	Interpretation
Configural	0.990	–	0.065	–	0.057	–	Good fit: same factorial structure in both groups.
Metric	0.989	−0.001	0.067	+0.002	0.060	+0.003	ΔCFI ≤ 0.01 → supports metric invariance.
Scalar	0.990	+0.001	0.062	−0.005	0.057	−0.003	ΔCFI ≤ 0.01 vs. metric → supports scalar invariance (thresholds for ordinal items).

CFI, Comparative Fit Index; RMSEA, Root Mean Square Error of Approximation; SRMR, Standardized Root Mean Square Residual; Δ, change from the previous model. Invariance was evaluated according to Chen (2007): ΔCFI ≤ 0.01 and ΔRMSEA ≤ 0.015 indicate no significant deterioration of model fit.

With scalar (threshold) invariance established (equal loadings and thresholds), latent-mean comparisons indicated that men scored lower than women on Stress (ΔM = −0.324, SE = 0.047, *p* < 0.001) and Anxiety (ΔM = −0.273, SE = 0.054, *p* < 0.001), with no difference on Depression (ΔM = 0.006, SE = 0.055, *p* = 0.908), under a standardized latent metric (std.lv = 1).

### 3.5 Differences in DASS-21 scores by previous diagnosis

As shown in Table 5, DASS-21 scores were compared between participants with and without a prior clinical diagnosis using the Mann–Whitney test. Statistically significant differences were observed across all dimensions (*p* < 0.001). The group with a prior diagnosis obtained higher mean scores in Stress (*M* = 10.82, *SD* = 5.03), Anxiety (*M* = 8.75, *SD* = 5.76), and Depression (*M* = 6.25, *SD* = 4.91), as well as in the total score (*M* = 25.82, *SD* = 14.23), compared to the group without a diagnosis (Stress: *M* = 7.26, *SD* = 4.60; Anxiety: *M* = 4.63, *SD* = 3.94; Depression: *M* = 3.97, *SD* = 3.83; Total: *M* = 15.86, *SD* = 11.18). The effect size, measured by rank-biserial correlation, was moderate for Stress (*r* = −0.40), Anxiety (*r* = −0.42), and the Total score (*r* = −0.41), and smaller for Depression (*r* = −0.29).

**Table 5:** Comparison of stress, anxiety, depression, and DASS-21 total scores according to the presence of a prior clinical diagnosis.

Variable	No diagnosis (*n* = 1,019) *M* (*SD*)	With diagnosis (*n* = 232) *M* (*SD*)	*U*	*p*	Rank-biserial *r*
Stress	7.26 (4.60)	10.82 (5.03)	70,666.5	< 0.001	−0.40
Anxiety	4.63 (3.94)	8.75 (5.76)	68,259	< 0.001	−0.42
Depression	3.97 (3.83)	6.25 (4.91)	83,946	< 0.001	−0.29
Total	15.86 (11.18)	25.82 (14.23)	68,916	< 0.001	−0.41

*M*, mean; SD, standard deviation; *U*, Mann–Whitney test statistic; *r*, rank-biserial correlation.

### 3.6 DASS-21 differences by self-reported emotional impairment

Differences in DASS-21 scores were analyzed between participants who did or did not report emotional impairment on two self-reported indicators. As shown in Table 6, participants who reported *“I did less than I wanted to due to emotional reasons”* obtained significantly higher scores in Stress (*U* = 81.76; *p* < 0.001; *r* = 0.45), Anxiety (*U* = 86.19; *p* < 0.001; *r* = 0.42), Depression (*U* = 87.67; *p* < 0.001; *r* = 0.40), and the Total score (*U* = 78.08; *p* < 0.001; *r* = 0.47), with moderate effect sizes.

**Table 6:** Comparison of DASS-21 scores according to self-reported emotional impairment indicators.

Emotional impairment indicator	Variable	No—*M* (*SD*)	Yes—*M* (*SD*)	*U*	*p*	Rank-biserial *r*
Did less than desired	Stress	6.92 (4.42)	10.86 (4.99)	81,76	< 0.001	0.45
	Anxiety	4.48 (4.00)	8.07 (5.26)	86,19	< 0.001	0.42
	Depression	3.63 (3.62)	6.61 (4.77)	87,67	< 0.001	0.41
	Total	15.03 (10.84)	25.54 (13.41)	78,08	< 0.001	0.47
Had to stop daily activities	Stress	7.32 (4.62)	11.99 (4.70)	41,26	< 0.001	0.52
	Anxiety	4.85 (4.25)	9.03 (5.28)	45,26	< 0.001	0.47
	Depression	3.95 (3.85)	7.30 (4.90)	48,04	< 0.001	0.44
	Total	16.12 (11.47)	28.31 (13.37)	40,59	< 0.001	0.53

*p*-values based on Mann–Whitney *U*-tests. Effect sizes correspond to rank-biserial correlations.

For the second indicator, *“I had to stop daily activities due to emotional reasons,”* significant differences were also found in Stress (*U* = 41.26; *p* < 0.001; *r* = 0.52), Anxiety (*U* = 45.26; *p* < 0.001; *r* = 0.47), Depression (*U* = 48.04; *p* < 0.001; *r* = 0.44), and the Total score (*U* = 40.59; *p* < 0.001; *r* = 0.53), with moderate-to-large effect sizes.

In both indicators, participants who responded affirmatively reported substantially higher scores on all DASS-21 dimensions, supporting the convergent validity of the instrument with recent self-reported emotional impairment.

### 3.7 Gender differences in DASS-21 scores

Between-group comparisons were conducted using Welch’s *t*-tests due to violations of normality and homoscedasticity. As shown in Table 7, women reported higher scores in Stress, Anxiety, and the Total DASS-21 than men, with small-to-moderate effect sizes (*Hedges’ g* = 0.31 [0.20, 0.43]). No significant difference was found for Depression. Descriptive estimates for the non-binary group (*n* = 5) are presented with 95% compatibility intervals to highlight the uncertainty associated with this small sample. Full exploratory analyses including this group are provided in Supplementary Table S12.

**Table 7:** Differences in DASS-21 scores according to gender.

Dimension	Male *M* (*SD*)	Female *M* (*SD*)	Non-binary *M* (*SD*)	95% compatibility interval (non-binary)	Welch’s *t* (df)	*p*	Hedges’ g [95% CI]
Stress	6.91 (4.48)	8.85 (5.05)	14.60 (3.85)	[9.82, 19.38]	7.14 (1205)	<0.001	0.41 [0.29, 0.52]
Anxiety	4.53 (3.91)	6.15 (5.04)	13.80 (3.03)	[10.04, 17.56]	6.30 (1142)	<0.001	0.36 [0.25, 0.47]
Depression	4.21 (3.96)	4.47 (4.24)	11.40 (5.90)	[4.08, 18.72]	1.13(1221)	0.26	0.06 [−0.05, 0.18]
Total	15.66 (13.13)	19.48 (11.13)	39.80 (12.38)	[24.43, 55.17]	5.51 (1187)	<0.001	0.31 [0.20, 0.426]

*M*, mean; SD, standard deviation. Welch’s *t*-tests were used due to violations of homogeneity of variances and normality. Hedges’ *g* effect sizes (with 95% confidence intervals) are reported for men–women comparisons. Descriptive results for the non-binary group (*n* = 5) are shown with compatibility intervals to reflect uncertainty due to small sample size.

### 3.8 DASS-21 and coping styles (PCI)

As theorized, DASS-21 scores showed negative associations of small-to-moderate magnitude with adaptive coping (Social Support, Reflective/Preventive, Proactive, Strategic Planning; *ρ* = −0.020 to −0.218) and moderate positive associations with Avoidant Coping (ρ = 0.245–0.418). The complete correlation matrix is presented in Table 8.

**Table 8:** Spearman correlations between DASS-21 dimensions and PCI factors, showing the association between emotional distress and coping styles.

Variable	Social support	Reflective/preventive coping	Proactive coping	Avoidant coping	Strategic planning
Stress	−0.059	−0.060	−0.183^***^	0.392^***^	−0.117^***^
Anxiety	−0.059	−0.020	−0.157^***^	0.375^***^	−0.102^***^
Depression	−0.202^***^	−0.088^**^	−0.218^***^	0.370^***^	−0.132^***^
Total	−0.110^***^	−0.058^*^	−0.207^***^	0.418^***^	−0.129^***^

Spearman’s ρ. Asterisks reflect Holm-adjusted significance within family (FWER-controlled): ^*^pHolm<0.05, ^**^pHolm<0.01, ^***^pHolm<0.001.

The overall pattern remained after multiplicity control: under the conservative Holm correction, all Proactive and Strategic Planning links (ρ ≈ −0.102 to −0.218) and all Avoidant links (ρ ≈ 0.245–0.418) were significant (pHolm<0.05), while the smallest effects for Social Support and Reflective/Preventive with Stress and Anxiety did not survive Holm (e.g., ρ ≈ −0.059 to −0.060; pHolm≈0.067–0.075). This profile aligns with the transactional stress–coping framework and provides convergent (maladaptive>adaptive) and discriminant validity evidence for the DASS-21.

## 4 Discussion

### 4.1 Explicit synthesis and hypothesis testing

The analyses supported the correlated three-factor structure of the DASS-21, revealing a predominant general distress factor and adequate reliability indices. Configural, metric, and scalar (threshold) invariance were demonstrated across gender, enabling valid comparisons of latent means. Associations with coping followed theoretically expected directions, and additional evidence was found for known-groups validity and associations with functional impairment.

### 4.2 Hypothesis outcomes

*H*1a (Three-factor structure): The DASS-21 exhibited a correlated three-factor model (stress, anxiety, depression) with acceptable global fit. *Outcome:* Supported.

*H*1b (General factor predominance): Second-order and bifactor evaluations indicated a prominent general distress factor (high *ECV*, *ωₕ*) with interpretable specific dimensions. *Outcome:* Supported.

*H*2 (Reliability): Internal consistency was adequate to excellent (*α*/*ω* ≥ 0.80 for subscales; ≥ 0.90 for the total scale), with narrow confidence intervals. *Outcome:* Supported.

*H*3 (Gender invariance): Configural, metric, and scalar (threshold) invariance held across women and men under the *WLSMV* framework (Δ*CFI* ≤ 0.01; Δ*RMSEA* ≤ 0.015). *Outcome:* Supported.

*H*3a (Latent mean differences; conditional on H3): Women scored higher on latent Stress and Anxiety (small effects), with no significant difference in Depression. *Outcome:* Supported.

*H*4 (Convergent validity with coping): Negative associations were observed with adaptive coping and positive associations with avoidant/evasive coping, the latter being the most robust. Familywise error was controlled with Holm correction. *Outcome:* Partially supported.

*H*5 (Known-groups validity): Students with a self-reported prior clinical diagnosis of anxiety or depression scored higher on the DASS-21 than those without such a diagnosis. *Outcome:* Supported.

*H*6 (Criterion-related validity: functional impairment): Higher DASS-21 scores were associated with greater recent functional impairment, with the total score showing the strongest correlation. *Outcome:* Supported.

### 4.3 Latent architecture (H1a–H1b) and the role of the general factor

The oblique three-factor CFA demonstrated good fit, and models incorporating a general factor (hierarchical and bifactor) further improved the structural representation of the DASS-21. Together with elevated *ECV* and *ωₕ* values, these results support essential unidimensionality, consistent with interpreting a total score while preserving the usefulness of subscales when they provide incremental validity (e.g., to guide domain-specific interventions). Recent national evidence obtained during the COVID-19 confinement also favored a bifactor solution with a strong general distress factor and three specific factors (Depression, Anxiety, Stress), converging on the presence of a substantive common component in Mexican adults (Salinas-Rodríguez et al., 2023).

At the same time, ESEM and bifactor–ESEM studies in young adults have proposed abbreviated forms (e.g., DASS-12) characterized by a dominant general factor and gender invariance, albeit with more limited external validity evidence and conducted outside university settings. In this context, the full three-factor solution retains interpretive value when it offers meaningful differentiation across domains and is supported by convergent and criterion validity evidence (Domínguez-Lara et al., 2024; see comparative discussion).

Early Mexican adaptation efforts reduced the scale to 14 items using exploratory procedures without invariance testing, underscoring the need for confirmatory and multigroup evaluations in subsequent research (Gurrola-Peña et al., 2006). ESEM was retained in the present study as a sensitivity analysis; when such models do not substantially outperform preferred confirmatory structures, they enhance methodological robustness without compromising the parsimony of the main model.

While the three-factor structure demonstrated strong empirical support in this study, it is important to acknowledge emerging methodological perspectives. From a network-analytic framework, Alvarenga et al. (2024) identified a potential additional factor, suggesting that symptom interconnections may at times transcend a strictly latent-variable perspective. Nevertheless, the confirmatory factor-analytic approach adopted here remains the most appropriate for testing measurement invariance and conducting latent-mean comparisons across groups, making it complementary to network-based formulations.

### 4.4 Reliability with confidence intervals (H2)

Estimates of Cronbach’s *α* and McDonald’s *ω* were consistent (*α/ω* ≥ 0.80 for subscales; ≈ 0.94 for the total scale), with narrow bootstrap confidence intervals, indicating measurement stability. This reliability pattern aligns with previous Mexican validations that reported adequate to high internal consistency across diverse contexts, including university and occupational samples, despite methodological variations (e.g., *EFA/CFA* with maximum likelihood and heterogeneous participants). Reporting confidence intervals for *α* and *ω*, and selecting estimators appropriate for ordinal indicators, represent recommended good practices in psychometric validation (Cruz-Flores et al., 2024; Salinas-Rodríguez et al., 2023).

### 4.5 Gender invariance with ordinal indicators (H3)

Using ordinal indicators and *WLSMV* estimation, configural, metric, and scalar (threshold) invariance were demonstrated across women (*n* = 607) and men (*n* = 629), with negligible changes in fit (e.g., Δ*CFI* ≤ 0.01; Δ*RMSEA* ≤ 0.015). Under this framework, latent means are comparable: men exhibited lower latent levels of Stress (Δ*M* = −0.324) and Anxiety (Δ*M* = −0.273), with no difference in Depression. Effect sizes were small. This pattern aligns with recent Mexican evidence from ESEM and bifactor–ESEM analyses reporting gender invariance and a prominent common distress component, even when shortened versions such as the DASS-12 were examined in young adults (Domínguez-Lara et al., 2024).

For ordinal data, scalar invariance implies equality of thresholds between groups; therefore, latent-mean differences reflect genuine variation in distress rather than response bias. Given the predominance of the general factor reported in recent Mexican studies (e.g., bifactor modeling in a national confinement sample) and the good three-factor fit observed in university populations, the total score is prioritized for gender comparisons, while subscales refine profiles when they contribute incremental validity (Salinas-Rodríguez et al., 2023).

Convergence with Domínguez-Lara et al. (2024) suggests that metric and scalar equivalence are attainable in Mexican samples when robust estimation frameworks (*ESEM/WLSMV*) and adequate sample sizes are employed. Nonetheless, prior university-based studies have been heterogeneous and often small in size, underscoring the importance of explicitly reporting the hierarchical testing flow (configural – metric - scalar), documenting Δ*CFI*, Δ*RMSEA*, and Δ*SRMR*, and considering partial invariance or alignment optimization when necessary.

### 4.6 Relations with coping (H4)

Consistent associations were observed between DASS-21 scores and coping styles in directions anticipated by theory; however, these effects were small to moderate and should be interpreted with caution regarding their practical significance. The pattern supports construct validity at a correlational level, yet it does not establish causal mechanisms. In particular, links with Avoidant/Evasive Coping—although the most robust—may partly reflect content proximity (e.g., avoidance, worry, rumination) to symptoms captured by Stress and Anxiety, raising the possibility of construct overlap as well as shared method variance from same-source, self-report data collected in a single session.

A second consideration is contextual and measurement constraints. Forced completion and the single-site setting may have attenuated variance in both distress and coping, while the age-homogeneous cohort could produce range restriction, depressing correlations for adaptive strategies (e.g., planning, proactive coping) even when theoretically relevant. Moreover, because estimates are cross-sectional, observed associations may index concurrent co-variation rather than directional influence (e.g., higher distress reducing the use of adaptive strategies, or elevated avoidance maintaining distress).

Taken together, the findings are compatible with interpreting the total DASS-21 score as an indicator of general distress and the subscales as potentially useful for profile-oriented guidance; nevertheless, incremental validity should be demonstrated explicitly before recommending subscale-driven intervention targets. Future work should (a) test whether subscales explain unique variance in academic or health outcomes beyond the total score, (b) use multi-method assessments of coping (e.g., behavioral or informant reports) to mitigate common-method bias, and (c) adopt longitudinal or cross-lagged designs to clarify temporal precedence. Stratified analyses (e.g., by gender, socioeconomic background, or prior diagnosis) may also illuminate whether the strength of distress–coping links varies across subgroups that are relevant for campus services.

### 4.7 Known-groups validity and functional impairment (H5–H6)

The DASS-21 successfully differentiated students who reported a prior diagnosis of anxiety or depression from those who did not (*H5*) and was associated with indicators of functional impairment during the previous week (e.g., *“did less than intended,” “stopped activities”*; *H6*). These results provide criterion-related validity evidence and underscore the ecological relevance of the scale in university contexts. The findings extend Mexican evidence by incorporating group comparisons and classification-oriented analyses (e.g., ROC), an approach that several earlier local validations either did not include or reported only partially (Salinas-Muñoz et al., 2024; Salinas-Rodríguez et al., 2023).

In university samples, Salinas-Muñoz et al. (2024) confirmed the three-factor structure and reliability of the DASS-21, but criterion validity was limited to correlational evidence, without clinical discrimination or ROC-based analyses. This constraint limited diagnostic interpretation and highlights the contribution of the present *H5–H6* analyses. In broader adult samples collected during the confinement period, Salinas-Rodríguez et al. (2023) favored a bifactor model and reported good internal consistency; however, the absence of clinical samples or cutoff derivations reinforces the need to examine external criteria and classification accuracy within specific subpopulations such as university students.

This study’s scope is limited by its single-site, private-university sample and cross-sectional design. Generalizability should therefore be verified in multi-site and longitudinal datasets. Clinical status was based on self-report, precluding diagnostic verification; thus, future cutoff points should be derived in clinically verified samples using ROC analyses against standardized interviews. Finally, the forced-completion setting of the digital platform eliminated missing data but may have introduced minor response-pressure artifacts.

### 4.8 Cross-cultural considerations and agenda

The present findings converge with Spanish-speaking and international validations regarding the three-factor structure and reliability values of ≥ 0.80, while adding evidence of scalar gender invariance using ordinal indicators. To consolidate local interpretability, future priorities include developing Mexican norms by age, sex, and institution type; estimating ROC-based cutoffs with clinically verified samples (including *AUC*, sensitivity/specificity, and predictive values with confidence intervals); and conducting multi-site, longitudinal studies to obtain test–retest reliability, verify invariance across institutions or academic semesters, and assess the incremental validity of subscales for academic and health-related outcomes. These recommendations align with the *Standards for Educational and Psychological Testing* (American Educational Research Association, American Psychological Association, and National Council on Measurement in Education, 2014) and the EFPA guidelines, and are reinforced by recent cross-cultural work emphasizing invariance and cultural adaptation in other languages and contexts (e.g., CES-D-8 and SWLS in Arabic; Ali et al., 2025a, 2025b).

Future research could also leverage secondary analyses of open databases to strengthen cross-validation and generalizability. For instance, publicly available datasets curated by Alvarenga et al. (2024) can be used to replicate the present findings in independent samples and to explore hybrid modeling approaches that integrate network and latent-variable perspectives. Such triangulation would help clarify when a general distress factor or a symptom-network topology offers the most informative framework for screening and profile-based applications.

## 5 Conclusions and recommendations

The DASS-21 exhibited a correlated three-factor structure with a predominant general distress factor and adequate-to-excellent reliability among Mexican university students. Configural, metric, and scalar (threshold) invariance were supported across women and men within an ordinal (*WLSMV*) framework, allowing valid latent-mean comparisons, indicating higher Stress and Anxiety in women and no significant gender difference in Depression.

Convergent associations with coping and links to functional impairment provide complementary external and criterion-related validity, supporting the use of the total score for general distress screening and the subscales for descriptive or profiling purposes.

Future research should prioritize the development of Mexican normative data and ROC-based clinical cutoffs using verified diagnostic samples, along with test–retest and multi-site invariance evaluations to consolidate the interpretability and applicability of the instrument across contexts.

## Data Availability

The original contributions presented in the study are included in the article/Supplementary material, further inquiries can be directed to the corresponding author.
