# Supplementary material for: Cross-sectional psychometric validation, convergent validity, and measurement invariance of the DASS-21 in Mexican university students
Source: Front Psychol. 2025 Dec 8;16:1707786. doi: 10.3389/fpsyg.2025.1707786 (PMC12719431; doi:10.3389/fpsyg.2025.1707786)
Supplement: Supplementary file 1 [file Data_Sheet_1.PDF]

Supplementary Material – Validation of the DASS-21 in Mexican University

Section A. Adaptation and Final Spanish Version

- Table S1. Original and adapted items of the DASS-21, with rationale for wording modifications, administration instructions, and scoring key.

Section B. Item- and Scale-Level Descriptive Statistics

- Table S2. Item-level descriptive statistics of the Mexican version of the DASS-21 (M, SD, skewness, kurtosis).
- Table S3. Item–total correlations and reliability indices (Cronbach's α, McDonald's ω) for the DASS-21 items.

Section C. Structural Models and Factor Loadings

- Table S4. Fit indices for the estimated models (CFA, hierarchical, bifactor, ESEM, and DASS-12).
- Table S5. Fully standardized loadings (λ) for the hierarchical CFA (second-order) model of the DASS-21.
- Table S6. Fully standardized loadings (λ) and bifactor indices (ECV, ωH, PUC) for the bifactor model of the DASS-21.
- Table S7. Standardized loadings (λ) for the ESEM solution (Geomin rotation).
- Table S8. Fully standardized loadings (λ) for the unidimensional DASS-12 short-form model.

Section D. Additional and Exploratory Analyses

- Table S10. Reliability indices for the DASS-12 short form (Cronbach's α, McDonald's ω, 95 % CI).
- Table S11. Reliability indices for the Proactive Coping Inventory (PCI).
- Table S12. Exploratory gender differences in DASS-21 scores (ANOVA results).

Table S1. Original and adapted items of the DASS-21 (Mexican university sample): source, Spanish candidate, final wording, and type of modification
Includes rationale summary, administration instructions, and scoring key.

Note. An asterisk (✱) indicates items included in the DASS-12 short form. "Type of modification" codes: LEX = lexical/simplification; IDM = idiomatic or cultural adjustment; CLAR = syntactic clarity/fluency; GEN = gender-inclusive language; CONS = terminological or tense consistency; SEM = semantic nuance.

#	Factor	Item (English)	Spanish (source/candidate)	Final (Spanish)	Change	Type
**1**	Stress	I found it hard to wind down	Encontré difícil bajarle a mi acelere	Encontré difícil calmarme/relajarme	Yes	IDM, CLAR
**2**	Anxiety	I was aware of dryness of my mouth	Estuve consciente de la resequedad en mi boca	—	No	—
**3**	Depression	I couldn't seem to experience any positive feeling at all	Al parecer no experimenté algún sentimiento positivo	—	No	—
**4**	Anxiety	I experienced breathing difficulty (e.g., …)	Experimenté dificultades al respirar (respiración muy rápida o quedarme sin aliento en ausencia de esfuerzo físico)	Tuve problemas para respirar (respiración muy rápida o falta de aire en ausencia de esfuerzo físico)	Yes	LEX, CLAR
**5✱**	Depression	I found it difficult to work up the initiative to do things	Encontré difícil tener la iniciativa para hacer las cosas	—	No	—
**6✱**	Stress	I tended to over-react to situations	Tendí a reaccionar exageradamente ante situaciones	Tendí a sobrerreaccionar ante situaciones	Yes	LEX, CONS
**7✱**	Anxiety	I experienced trembling (e.g., in the	Experimenté temblores (por ejemplo, en las manos)	—	No	—

		hands)				
**8**	Stress	I felt that I was using a lot of nervous energy	Sentí que tenía muchos nervios	Sentí que tuve nervios	Yes	CONS
**9✱**	Anxiety	I was worried about situations in which I might panic and make a fool of myself	Estuve preocupado acerca de las situaciones ante las cuales me asusto y hago el ridículo	Me preocupé por situaciones que me asustan y puedo quedar en ridículo	Yes	CLAR
**10✱**	Depression	I felt that I had nothing to look forward to	Sentí que no tenía nada que esperar	—	No	—
**11✱**	Stress	I found myself getting agitated	Me encontré a mí mismo poniéndome nervioso	Me di cuenta de que me ponía nerviosa/o	Yes	GEN, CLAR
**12✱**	Stress	I found it difficult to relax	Encontré difícil relajarme	—	No	—
**13**	Depression	I felt down-hearted and blue	Me sentí desanimado y triste	—	No	—
**14✱**	Stress	I was intolerant of anything that kept me from getting on with what I was doing	Estuve intolerante con todo lo que me distrajera de lo que estaba haciendo	—	No	—
**15✱**	Anxiety	I felt I was close to panic	Sentí que estuve a punto de entrar en pánico	—	No	—
**16✱**	Depression	I was unable to become	Me sentí incapaz de sentirme entusiasmado	Fui incapaz de entusiasmarme por	Yes	LEX, CLAR

		enthusiastic about anything	acerca de algo	algo		
**17✱**	Depression	I felt I wasn't worth much as a person	Sentí que no valía como persona	—	No	—
**18**	Stress	I felt that I was rather touchy	Sentí que estaba muy irritable	—	No	—
**19**	Anxiety	I was aware of the beating of my heart in the absence of physical exertion (e.g., …)	Sentí los latidos de mi corazón a pesar de no haber hecho esfuerzo físico (ejemplo: sentir aumento del ritmo cardiaco o que va más lento)	—	No	—
**20✱**	Anxiety	I felt scared without any good reason	Sentí miedo sin alguna razón	—	No	—
**21**	Depression	I felt that life was meaningless	Sentí que la vida no tenía sentido	Consideré que la vida no tenía sentido	Yes	SEM

Summary of major modifications

- **IDM/CLAR (Item 1): Replaced a colloquial expression ("bajarle a mi acelere") with a neutral, clinically equivalent phrase ("calmarme/relajarme").**
- **LEX/CLAR (Items 4, 9, 16): Simplified and modernized wording while preserving semantic equivalence.**
- **GEN (Item 11): Introduced gender-inclusive language ("nerviosa/o").**
- **CONS (Items 6, 8): Harmonized tense and terminology across items.**
- **SEM (Item 21): Shifted from "felt" to "considered" to express reflective judgment without altering core meaning.**

Administration instructions

- **Reference period: Past week.**
- **Response format: 0 = *Did not apply to me at all*; 1 = *Applied to me to some degree or some of the time*; 2 = *Applied to me to a considerable degree or a good part of time*; 3 = *Applied to me very much or most of the time*.**
- **Instruction to participants:"Below are statements about experiences over the past week. Please circle the option that best describes how much each statement applied to you."**

Scoring key

- **Subscales:**
  - ***Stress:* Items 1, 6, 8, 11, 12, 14, 18**
  - ***Anxiety:* Items 2, 4, 7, 9, 15, 19, 20**
  - ***Depression:* Items 3, 5, 10, 13, 16, 17, 21**
- **Computation: Sum the seven items per subscale (range = 0–21). Total DASS-21 = sum of all 21 items (range = 0–63).**
- **Reverse-scored items: None.**

Section B. Item- and Scale-Level Descriptive Statistics

Table S2. Item-level descriptive statistics of the Mexican version of the DASS-21

Means, standard deviations (SD), skewness, and kurtosis for the 21 items of the Mexican adaptation of the DASS-21 (n = 1,251).

Item	Mean	SD	Skewness	Kurtosis
1. Encontré difícil calmarme/relajarme	1.1	0.8	0.5	-0.2
2. Estuve consciente de la resequedad en mi boca	1.1	1.0	0.4	-0.9
3. Al parecer no experimenté algún sentimiento positivo	0.6	0.8	1.2	0.8
4. Tuve problemas para respirar (respiración muy rápida o falta de aire en ausencia de esfuerzo físico)	0.5	0.8	1.5	1.6
5. Encontré difícil tener la iniciativa para hacer las cosas	1.0	0.9	0.6	-0.3
6. Tendí a sobrerreaccionar ante situaciones	1.0	0.9	0.7	-0.5
7. Experimenté temblores (por ejemplo, en las manos)	0.7	0.9	1.2	0.3
8. Sentí que tuve nervios	1.5	0.9	0.1	-0.9
9. Me preocupé por situaciones que me asustan y puedo quedar en ridículo	1.2	1.0	0.4	-1
10. Sentí que no tenía nada que esperar	0.7	0.9	1.2	0.5
11. Me di cuenta de que me ponía nerviosa/o	1.5	1.0	0.2	-0.9
12. Encontré difícil relajarme	1.0	0.9	0.6	-0.3
13. Me sentí desanimado y triste	0.9	0.9	0.7	-0.2
14. Estuve intolerante con todo lo que me distrajera de lo que estaba haciendo	0.9	0.9	0.7	-0.4
15. Sentí que estuve a punto de entrar en pánico	0.5	0.9	1.5	1.2
16. Fui incapaz de entusiasmarme por algo.	0.6	0.9	1.4	1.0
17. Sentí que no valía como persona	0.4	0.8	1.9	2.9
18. Sentí que estaba muy irritable	1.0	1.0	0.7	-0.7
19. Sentí los latidos de mi corazón a pesar de no haber hecho esfuerzo físico (Ejemplo: Sentir aumento del ritmo cardiaco o que va más lento).	0.7	1.0	1.2	0.3
20. Sentí miedo sin alguna razón	0.7	0.9	1.2	0.4
21. Consideré que la vida no tenía sentido	0.3	0.7	2.6	6.1

Note.

Skewness and kurtosis values were computed using the sample formulas. Positive skewness indicates right-tailed distributions (more responses at the lower end of the scale). Item 21 shows marked positive skewness and kurtosis, reflecting low endorsement of severe depressive content, as expected in non-clinical university sample

Table S3. Item–total correlations and reliability indices (Cronbach's α, McDonald's ω, item-rest correlations)

McDonald's ω, Cronbach's α, and corrected item–total correlations for each item. "If item dropped" values indicate the reliability of the total score when that item is removed (n = 1,251)

Item	If item dropped: McDonald's ω	If item dropped: Cronbach's α	Item-rest correlation
1. Encontré difícil calmarme/relajarme	.94	.93	.62
2. Estuve consciente de la resequedad en mi boca	.94	.94	.38
3. Al parecer no experimenté algún sentimiento positivo	.94	.94	.48
4. Tuve problemas para respirar (respiración muy rápida o falta de aire en ausencia de esfuerzo físico)	.94	.94	.60
5. Encontré difícil tener la iniciativa para hacer las cosas	.94	.94	.54
6. Tendí a sobrerreaccionar ante situaciones	.94	.94	.60
7. Experimenté temblores (por ejemplo, en las manos)	.94	.94	.61
8. Sentí que tuve nervios	.94	.93	.67
9. Me preocupé por situaciones que me asustan y puedo quedar en ridículo	.94	.93	.66
10. Sentí que no tenía nada que esperar	.94	.93	.64
11. Me di cuenta de que me ponía nerviosa/o	.94	.93	.69
12. Encontré difícil relajarme	.93	.93	.74
13. Me sentí desanimado y triste	.94	.93	.70
14. Estuve intolerante con todo lo que me distrajera de lo que estaba haciendo	.94	.93	.64
15. Sentí que estuve a punto de entrar en pánico	.93	.93	.72
16. Fui incapaz de entusiasmarme por algo.	.94	.94	.58
17. Sentí que no valía como persona	.94	.93	.64
18. Sentí que estaba muy irritable	.94	.93	.68
19. Sentí los latidos de mi corazón a pesar de no haber hecho esfuerzo físico (Ejemplo: Sentir aumento del ritmo cardiaco o que va más lento).	.94	.93	.69
20. Sentí miedo sin alguna razón	.94	.93	.70
21. Consideré que la vida no tenía sentido	.94	.94	.55

Notes.

1. Cronbach's α and McDonald's ω reflect total-score reliability across all 21 items; values above .90 indicate excellent internal consistency for screening purposes.

2. Removing any single item did not substantially increase reliability, suggesting that all items contribute meaningfully to the total DASS-21 score.

Section C. Model Fit and Factor Loadings

Table S4. Fit indices for the estimated models (CFA, hierarchical, bifactor, ESEM, and DASS-12)

Comparative goodness-of-fit indices for all tested models.

Model	χ^2(df)	CFI	TLI	RMSEA [90% CI]	SRMR	Estimator	Rotation
Three-factor CFA (oblique)	1249.35 (186)***	0.989	0.988	0.068 [0.064–0.071]	0.052	DWLS	None
Hierarchical CFA	–	0.990	0.988	0.068 [0.064–0.071]	0.052	DWLS	–
Bifactor CFA	–	**0.997**	**0.996**	**0.022 [0.019–0.025]**	**0.037**	DWLS	–
ESEM (Geomin rotation)	1922.97 (186)***	0.990	0.988	0.068 [0.064–0.071]	0.052	DWLS	Geomin
DASS-12 (unidimensional)	–	0.997	0.995	0.026 [0.022–0.030]	0.035	DWLS	–

Notes.

- CI = confidence interval; DWLS = diagonally weighted least squares; ESEM = exploratory structural equation modeling.
- All χ^2 tests significant at $p < .001$.
- CFI ≥ .95, TLI ≥ .95, RMSEA ≤ .05, and SRMR ≤ .08 indicate excellent fit; RMSEA values up to .08 are considered acceptable (Hu and Bentler, 1999; Brown, 2015).
- The three-factor oblique model showed adequate fit, while bifactor and hierarchical models demonstrated superior fit, supporting the presence of a strong general distress factor with meaningful specific dimensions.

Table S5. Fully standardized factor loadings (λ) for the three-factor oblique model of the DASS-21

Standardized loadings with 95% robust CIs, z, and p for Stress, Anxiety, and Depression factors.

Factor	Item	λ	95% CI	z	p
**Stress**	S1	0.57	[0.53–0.61]	25.9	< .001
	S6	0.58	[0.53–0.63]	23.1	< .001
	S8	0.69	[0.65–0.73]	33.2	< .001
	S11	0.72	[0.68–0.76]	36.4	< .001
	S12	0.70	[0.66–0.74]	32.0	< .001
	S14	0.61	[0.56–0.66]	24.7	< .001
	S18	0.70	[0.65–0.75]	28.1	< .001
**Anxiety**	A2	0.38	[0.33–0.44]	13.7	< .001

	A4	0.50	[0.45–0.55]	18.3	< .001
	A7	0.62	[0.56–0.67]	21.7	< .001
	A9	0.69	[0.64–0.74]	28.2	< .001
	A15	0.67	[0.62–0.72]	25.5	< .001
	A19	0.71	[0.65–0.76]	26.1	< .001
	A20	0.70	[0.64–0.75]	25.3	< .001
**Depression**	D3	0.42	[0.37–0.47]	17.0	< .001
	D5	0.51	[0.46–0.56]	20.1	< .001
	D10	0.62	[0.57–0.68]	23.7	< .001
	D13	0.64	[0.60–0.69]	28.8	< .001
	D16	0.58	[0.54–0.64]	22.9	< .001
	D17	0.58	[0.52–0.64]	19.9	< .001
	D21	0.46	[0.40–0.52]	14.3	< .001

Notes.

- All factor loadings are standardized and significant at $p < .001$.
- DWLS estimator with robust standard errors was used (ordered-categorical indicators).
- CI = 95% confidence interval.
- Item A2 ($\lambda = .38$) shows a moderate/low loading, consistent with an autonomic symptom less central in non-clinical samples.

Table S6. Fully standardized loadings (λ) for the hierarchical CFA (second-order) model of the DASS-21

Item	Second order	Fist order			
		Stress	Anxiety	Depression	95% CI
Stress	0.94				[0.92 – 0.96]
Anxiety	0.97				[0.95 – 0.99]
Depression	0.86				[0.83 – 0.89]
S1		0.76			[0.72 – 0.79]
S6		0.68			[0.63 – 0.72]
S8		0.83			[0.80 – 0.86]
S11		0.86			[0.83 – 0.88]
S12		0.87			[0.84 – 0.89]
S14		0.74			[0.70 – 0.78]
S18		0.78			[0.75 – 0.81]

A2		0.44		[0.38 – 0.49]
A4		0.72		[0.69 – 0.77]
A7		0.73		[0.69 – 0.77]
A9		0.79		[0.75 – 0.82]
A15		0.87		[0.84 – 0.90]
A19		0.80		[0.77 – 0.84]
A20		0.81		[0.78 – 0.85]
D3			0.63	[0.57 – 0.68]
D5			0.66	[0.61 – 0.70]
D10			0.79	[0.75 – 0.82]
D13			0.84	[0.81 – 0.87]
D16			0.75	[0.71 – 0.80]
D17			0.87	[0.84 – 0.90]
D21			0.81	[0.75 – 0.86]

Note. A higher-order general distress factor explaining shared variance among first-order Stress, Anxiety, and Depression factors; ordered-categorical indicators estimated with DWLS.

Table S7. Fully standardized loadings (λ) for the bifactor model of the DASS-21

General distress (G) and group factors (Stress, Anxiety, Depression); ordered-categorical indicators, DWLS.

Item	G	Stress	Anxiety	Depression	95% CI (G)
S1	0.54	0.02	--	--	[0.53–0.56]
S6	0.59	-0.12	--	--	[0.57–0.61]
S8	0.67	0.42	--	--	[0.64–0.69]
S11	0.71	0.44	--	--	[0.69–0.73]
S12	0.69	0.02	--	--	[0.67–0.71]
S14	0.63	-0.17	--	--	[0.61–0.66]
S18	0.72	-0.19	--	--	[0.70–0.74]
A2	0.38	--	0.10	--	[0.36–0.40]

A4	0.45	--	0.29	--	[0.43–0.47]
A7	0.56	--	0.31	--	[0.54–0.59]
A9	0.73	--	-0.11	--	[0.71–0.76]
A15	0.63	--	0.19	--	[0.61–0.65]
A19	0.64	--	0.46	--	[0.61–0.67]
A20	0.66	--	0.15	--	[0.64–0.69]
D3	0.34	--	--	0.27	[0.32–0.36]
D5	0.46	--	--	0.22	[0.44–0.49]
D10	0.54	--	--	0.29	[0.52–0.57]
D13	0.59	--	--	0.25	[0.57–0.61]
D16	0.46	--	--	0.42	[0.43–0.48]
D17	0.46	--	--	0.36	[0.44–0.49]
D21	0.34	--	--	0.32	[0.32–0.36]

Notes.

All loadings are standardized and significant at $p < .001$. DWLS estimator with robust SEs was used (ordered-categorical indicators). CI = 95% confidence interval.

ECV = proportion of common variance explained by the general factor; ωH = reliability of the general factor; PUC = percentage of correlations influenced only by the general factor.

Values of ECV ≥ .70 and ωH ≥ .80 indicate an essentially unidimensional structure with meaningful specific factors (Rodriguez et al., 2016)

Table S8. Standardized loadings (λ) for the ESEM solution (Geomin rotation)

item	Stress λ	Anxiety λ	Depression λ	h²	95% CI
E1	**0.78**			0.63	[0.74 – 0.81]
A2	0.36		**0.82**	0.91	[0.82 – 0.82]
D3	**0.44**	0.35		0.43	[0.37 – 0.52]
A4	**0.7**			0.51	[0.65 – 0.75]
D5	**0.53**			0.41	[0.47 – 0.59]
E6	**0.64**			0.44	[0.59 – 0.69]
A7	**0.77**	–0.14		0.52	[0.72 – 0.82]
E8	**0.99**	–0.52	–0.33	0.82	[0.90 – 1.00]
A9	**0.87**	–0.25	–0.29	0.64	[0.82 – 0.93]
D10	**0.68**			0.57	[0.64 – 0.73]
E11	**0.99**	–0.45	–0.34	0.81	[0.91 – 1.00]
E12	**0.76**			0.57	[0.76 – 0.76]
D13	**0.7**			0.65	[0.65 – 0.70]
E14	**0.66**			0.54	[0.61 – 0.71]
A15	**0.87**			0.76	[0.83 – 0.91]
D16	**0.54**	0.42		0.62	[0.48 – 0.61]
D17	**0.68**	0.39	–0.08	0.79	[0.62 – 0.73]
E18	**0.71**			0.59	[0.66 – 0.75]
A19	**0.81**			0.62	[0.77 – 0.86]
A20	**0.84**	–0.10	–0.12	0.64	[0.79 – 0.89]
D21	**0.56**	0.41		0.64	[0.56 – 0.56]

Note.

Standardized factor loadings ($|\lambda| \geq .30$) from the oblique three-factor exploratory structural equation model (ESEM) are displayed. Blank cells indicate loadings < .30 in absolute value. Primary factor loadings are bolded (or identified in the "Primary" column). Cross-loadings (i.e., an item loading ≥ .30 on more than one factor) are shown in the same row. The three factors were interpreted as Stress, Anxiety and Depression based on item content and dominant loadings. Inter-factor correlations were r = .32 (Stress–Anxiety), r = .17 (Stress–Depression), and r = –.27 (Anxiety–Depression). The model was estimated using diagonally weighted least squares (DWLS) with polychoric correlations, assuming ordinal item responses (4-point Likert scale). An oblique Geomin rotation (ε = 0.5) was applied. The ESEM converged in 58 iterations, with all cross-loadings freely estimated and factor variances fixed to 1.0 for identification. Negative loadings reflect inverse associations with the latent factor but do not indicate reverse-scored items, as all DASS items are positively worded.

Table S9. Fully standardized loadings (λ) for the unidimensional DASS-12 model

Factor	Item	λ	95% CI
**Stress**	S6	0.57	[0.52 – 0.62]

	S11	0.71	[0.66 – 0.74]
	S12	0.67	[0.62 – 0.72]
	S14	0.60	[0.56 – 0.65]
**Anxiety**	A7	0.57	[0.52 – 0.63]
	A9	0.74	[0.70 – 0.79]
	A15	0.64	[0.59 – 0.69]
	A20	0.69	[0.62 – 0.73]
**Depression**	D5	0.53	[0.47 – 0.59]
	D10	0.65	[0.59 – 0.70]
	D16	0.55	[0.50 – 0.60]
	D17	0.56	[0.52 – 0.61]

Section D.

Table S10. Reliability indices for the DASS-12 short form (Mexican university sample)

Cronbach's α and McDonald's ω with 95 % confidence intervals (CIs) for each subscale (n = 1251).

Subscale	Cronbach's α [95% CI]	McDonald's ω [95% CI]
Stress	.79 [.77-.80]	.79 [.77–.81]
Anxiety	.80 [.78-.82]	.80 [.78–.82]
Depression	.77 [.75-.79]	.77 [.75–.79]

Notes.

- Reliability estimates were obtained from polychoric correlations using a DWLS estimator and bootstrapped 95 % CIs (1,000 replications).
- Values ≥ .70 indicate acceptable internal consistency for research use, and values ≥ .80 are considered good for applied screening (Nunnally and Bernstein, 1994).
- DASS-12 subscales correspond to the highest-loading items of the DASS-21.
- The 12 items correspond to the short-form configuration proposed by Domínguez-Lara (2022), representing the strongest indicators of Stress, Anxiety, and Depression.

Table S11. Reliability indices for the Proactive Coping Inventory (PCI)

Subscale	Cronbach's α [95% CI]	McDonald's ω [95% CI]
Strategic	.70 [.62–.75]	.75 [.69–.81]
Avoidance	.77 [.69–.88]	.79 [.71–.90]
Instrumental/Emotional Support	.90 [.86–.95]	.90 [.87–.96]
Proactive	.87 [.83–.96]	.88 [.83–.97]
Reflective/Preventive	.89 [.85–.96]	.89 [.85–.96]

Note. Cronbach's α and McDonald's ω with 95 % confidence intervals (CIs) for each PCI subscale (n =1,251).

Section D. Additional and Exploratory Analyses

Table S12. Exploratory gender differences in DASS-21 scores

One-way ANOVAs exploring mean differences in Stress, Anxiety, and Depression across gender groups (male, female, non-binary). Given the small size of the non-binary subgroup (n = 5), results are considered exploratory and descriptive only.

Dimension	F(df1, df2)	p	ω²	Male M (SD)	Female M (SD)	Non-binary M (SD)	Post hoc comparisons
Stress	30.60 (2, 1238)	< .001	.046	6.91 (4.48)	8.85 (5.05)	14.60 (3.85)	M < F, M < NB, F < NB
Anxiety	28.90 (2, 1238)	< .001	.043	4.53 (3.91)	6.15 (5.04)	13.80 (3.03)	M < F, M < NB, F < NB
Depression	7.99 (2, 1238)	< .001	.011	4.21 (3.96)	4.47 (4.24)	11.40 (5.90)	M ≈ F, M < NB, F < NB

Notes.
M = mean; *SD* = standard deviation; ω² = omega squared (effect size).
Post hoc comparisons were conducted using Tukey's HSD test with *p*-values adjusted for multiple comparisons.
Results for the non-binary group (*n* = 5) are presented descriptively and should not be generalized.
Although the overall pattern suggests higher emotional distress among non-binary participants, these findings are preliminary and intended solely as an exploratory complement to the main analyses.
